# Veterinary Science and the United States Army

**Published:** 1893-02

**Authors:** R. W. Shufeldt

**Affiliations:** Med. Dept., U. S. A.


### COMMUNICATIONS.

### VETERINARY SCIENCE AND THE UNITED STATES ARMY.

(*A Rejoinder to Dr. J. P. Turner.*)

By R. W. Shufeldt, Med. Dept. U. S. A.

Under the title of "A Plea for the More Thorough Study of the Structure of the Horse," the present writer contributed last year a paper to this Journal (Dec., 1892, pp. 758, 759) wherein he pointed out an ignorance of equine osteology in certain quarters, and extended his remarks to include officers of several departments of our Army.

This was replied to by Dr. J. P. Turner, Veterinarian of the Sixth U. S. Cavalry, in the succeeding issue of the Journal, in a letter wherein he attempts to disprove my statements and place the state of affairs in the light in which he sees it (Jan., 1893, pp. 44–46).

Now, Dr. Turner appears to be laboring under the impression that the writer is one of those "relics of the days of the empire in the army," and has seen nothing of its veterinary department since a "past decade." Let me, in the first place, disabuse his mind of this illusion, and do so by stating that I have only very recently been placed upon the "retired list," and that only five years ago, I served at the headquarters of the very cavalry regiment of which he is now veterinarian, and that I also served at at a remoter period for several years at the headquarters of the Fifth U. S. Cavalry, the Third U. S. Cavalry, and as we have but ten regiments representing that arm of the service in our army, it is just possible I may not be as illy informed in the premises as he seems to imagine. Moreover, I would have him know that I have always been friendly to the best interests of his department, and in support of this statement I would kindly invite his attention to a paper of mine entitled, "The Veterinary Service of the United States Army," published in this Journal a number of years ago (Vol. VIII, Apr.,

1887, pp. 138–143). That paper had a good effect, and even received the praise of the most inefficient Secretary of War this country has ever seen—the Hon. W. C. Endicott.

I have been told by one of the best of veterinarians that that article was largely instrumental in inviting the attention of the War Office to the deplorable condition of the veterinary department of our Army as it existed at the time the paper appeared.

It is somewhat amusing to observe how it is that Dr. Turner, in his reply to my December communication, in endeavoring to combat my statements, in reality agrees with them in the main.

I deplore the fact of the scarcity of really good veterinary colleges in this country, and he replies in the statement that "To anyone who has closely studied the facilities for teaching anatomy in some of our veterinary colleges, the natural results are not surprising, especially where the veterinarian has learned his anatomy entirely [sic] from highly colored plates and Auzoux models, so that in many incidences we agree with this gentleman in his conclusions." Now, it was against the very class of colleges to which he refers that my remarks were directed, as the title and drift of my article will vouch for. That we have a number of excellent ones there can be no question.

When Dr. Turner says that "Luckily in these days we now see the army veterinarian in his proper place, both officially and socially, in many of our best cavalry regiments," I beg to differ with him. In the first place they are not officially recognized in the *Army Register* as a department of commissioned officers, and they will never occupy the *social position* that they should until they are. My ideas upon this subject are fully expressed in my article on "The Veterinary Service in the U. S. Army," named above.

For one I regret to see so capable a veterinarian, as my critic appears to be, so satisfied and complaisant in his now-position, for I am of those who believe in the co-equality of the sciences, be they veterinary or human. In other words, veterinary anatomy or pathology means just as much to me as human anatomy or human pathology. It requires quite as much ability to comprehend the structure of a horse as it does the structure of one of our own species, and those subjects should be studied in the same exhaustive manner.

Dr. Turner takes exception to my statement wherein I said that "With the rarest exception the government veterinarian in the army knows little or nothing of the subject of equine anatomy, and this stricture may with equal truth be extended to include the

West Point graduates in the artillery and cavalry arms of the service," and, yet, almost in the same breath, replies by the statement that "It is nevertheless true that the ordinary, recently graduated West-Pointer is comparatively ignorant of equine anatomy, yet these intelligent young officers cannot be blamed for this failing, since they receive no instruction whatever in this branch at the Military Academy," and, further, it would appear to be a mere *matter of luck* that the cavalry officer is *ever* to know anything of the subject, and "for the artillery officer there is no such chance, since our Government fails to provide artillery with the services of a veterinarian." (!) The practice he alludes to of Army veterinarians delivering lectures to cavalry officers during the winter in garrison is a step in the right direction, but such instruction should begin far earlier in the cavalry officer's career—that is, at West Point. This is the *point* that I insisted upon in my communication, and the Government will do well to take advantage of this "pointer."

I fail to see the pertinency of Dr. Turner's question as to whether it would "be showing much wisdom and respect if I were to condemn the entire Medical Department of the U. S. Army, which department is one of the best organized military staffs in existence, and consists of scientific and refined officers, simply because I have met officers of this corps who could talk far more intelligently about ornithology, botany and palæontology than about human anatomy and kindred subjects?"—and, moreover, I would add that so far as life has thus far carried me, I have yet to meet a department, anywhere, that was *perfect* in its organization, and that the Medical Department of the Army is wholly composed "of scientific and refined officers" is a statement quite open to question.

In conclusion let me say that Dr. Shufeldt feels that he has the right "to intrude into a territory," be it one where it is "extremely delicate for him to dwell in" it or not. So long as he enters there with the but one purpose, and that is with the intention of improving that territory by what he has to say about it. If I can print anything that may tend to further the interests of veterinary science in this country and point out its needs, or even its defects as I see them, you may rest assured that such opportunity will not be neglected.

